# The Protective Function and Modification of Secondary Metabolite Accumulation in Response to Light Stress in *Dracocephalum forrestii* Shoots

**DOI:** 10.3390/ijms22157965

**Published:** 2021-07-26

**Authors:** Izabela Weremczuk-Jeżyna, Katarzyna Hnatuszko-Konka, Liwia Lebelt, Izabela Grzegorczyk-Karolak

**Affiliations:** 1 Department of Biology and Pharmaceutical Botany, Faculty of Pharmacy, Medical University of Lodz, Muszyńskiego 1, 90-151 Lodz, Poland; izabela.grzegorczyk@umed.lodz.pl; 2 Department of Molecular Biotechnology and Genetics, Faculty of Biology and Environmental Protection, University of Lodz, Banacha 12/16, 90-237 Lodz, Poland; katarzyna.hnatuszko@biol.uni.lodz.pl; 3 Bioorganic Chemistry Laboratory, Faculty of Pharmacy, Medical University of Lodz, Muszyńskiego 1, 90-151 Lodz, Poland; liwia.lubowiecka@umed.lodz.pl

**Keywords:** apigenin derivative, fluorescent light, light conditions, LEDs, rosmarinic acid, salvianolic acid B, shoot culture

## Abstract

The aim of this work was to determine the effect of stress conditions caused by different light sources, i.e., blue LED (λ = 430 nm), red LED (λ = 670 nm), blue and red LED (70%:30%) and white LED (430–670 nm) on the growth and morphology of cultivated in vitro *Dracocephalum forrestii* shoot culture. It also examines the effects on bioactive phenolic compound production and photosynthetic pigment content, as well as on antioxidant enzyme activity (CAT, SOD, POD) and antioxidant properties. The most beneficial proliferation effect was observed under white LEDs (7.1 ± 2.1 shoots per explant). The white and blue lights stimulated the highest fresh weight gain, while red light induced the highest dry weight gain. The total phenolic acid content ranged from 13.824 ± 1.181 to 20.018 ± 801 mg g DW^−1^ depending on light conditions. The highest content of rosmarinic acid was found in the control shoots (cultivated under fluorescent lamps), followed by culture grown under red light. All LED treatments, especially red and blue, increased salvianolic acid B content, and blue increased apigenin *p*-coumarylrhamnoside biosynthesis. The greatest ferric reduction activity was observed in shoots cultivated under red light, followed by blue; this is associated with the presence of the highest total phenol content, especially phenolic acids. Similarly, the highest DPPH radical scavenging potential was observed under red light followed by blue. This study proves that LEDs have emerged as significant support for directed in vitro propagation, taking advantage of specific stress responses on various light spectra. This study also showed how stress induced by different LED light spectra increases in *Dracocephalum forrestii* the synthesis of pharmacologically-active compounds. Hence, light stress may turn out to be a simpler alternative to metabolic engineering for improving the production of secondary metabolites of therapeutic value.

## 1. Introduction

The production and accumulation of secondary metabolites (SM) is an example of a sophisticated process developed by plants to facilitate survival and adaptation. This heterogeneous group of compounds is involved in defence against abiotic and biotic stresses, as well as in signalling of symbiotic communication, attracting pollinating animals and protecting plants from UV radiation and oxidants [1]. A range of classifications exist for plant metabolites. They may refer to chemical structure (concerning presence of sugars or rings), solubility in water or organic solvents, composition (presence of nitrogen element) and their biosynthetic pathway [1]. However, the most practical classification may be that based on synthetic route, which results in three large classes: terpenes, phenolic compounds and alkaloids. Of these, the most abundant group is the terpenes, followed by the phenolic compounds [1]. The latter class is synthesized in plant cells by the shikimate/phenylpropanoid and/or the malonate/acetate pathways. Phenolic compounds are chemically a very diverse group that encompass several thousand SM in higher plants but are uncommon in bacteria, fungi and algae. They are considered to play a crucial role in the antioxidative defence system by neutralising free radicals and other oxidative agents released under environmental stress conditions, typically high light level, nutrient deficiency, low temperatures and pathogen infection. A body of evidence suggests that plant phenolics support the capability of plants to scavenge reactive oxygen species (ROS) [2,3].

In addition to their vital role in maintaining plant fitness and competitiveness, SM also serve as food additives or important pharmaceuticals. Natural compounds, and SM in particular, can provide effective and safe alternatives to synthetic drugs for human therapy. It has been estimated that one fourth of the prescribed drugs contain components directly or indirectly derived from plants [4]. One plant species that has been screened for pharmacologically bioactive molecules is the Tibetan medical species *Dracocephalum forrestii* W.W. Smith, which grows in Chinese mountains in Yunnan province, where its aerial parts are known for their astringent, diuretic and antipyretic properties [5]. Among its SM, terpenoids, flavonoids, phenolic acids and lignans are believed to predominate [6]. However, most of the therapeutic activities of *Dracocephalum* result from the synthesis and accumulation of phenolic compounds, including a number of caffeic acid derivatives (e.g., chlorogenic acid, rosmarinic acid, salvianolic acid B). Interestingly, transgenic hairy roots have also been found to demonstrate antioxidant, anti-inflammatory and anticancer properties [7].

However, the chemical synthesis of plant compounds is often expensive and not economically viable due to their complex nature; in addition, metabolic engineering requires a good theoretical understanding of SM pathways. With this in mind, there is a need to find a procedure to increase the production of SM in *D. forrestii*. The synthesis of SM can be accelerated using factors known as elicitors, also commonly defined as stressors [8]. One such stressor known to influence the growth and morphology of shoots is the choice of light spectra, which may also influence the production of bioactive phenolic compounds. Light influences and alters plant physiological pathways in varied ways by affecting the sophisticated system of photoreceptors developed by plants. It can influence the processes behind flowering, circadian rhythm, photosynthesis, production of carotenoids and anthocyanins, vegetative growth, shoot biomass, production of different defence proteins or decreased leaf area. However, the nature of the feedback is strongly species and/or cultivar dependent [9,10,11].

Therefore, the aim of the present study is to demonstrate the influence of light as a stressor on the growth and phytochemical profile of *D. forrestii*, particularly on the accumulation of secondary compounds with therapeutic value. The study examines the effect of different light spectra, induced by LED (Light Emitting Diode) lamps, on tissue cultures of *D. forrestii*. Four types of LED are used: blue light λ = 430 nm; red light λ = 670 nm; a combination of red and blue lights—70%:30%, and white light λ ∈ (430–670 nm). Fluorescent light was used as a reference. Although it is well known that light affects plant metabolism, growth and development, the nature of the response varies between species, and no such information is currently available for the genus *Dracocephalum*. Moreover, most previous studies on the effect of light as a stressor have examined ornamental species and focused on growth and morphological traits. A relatively low number of studies have been related to light-induced changes in secondary metabolism associated with the production of plant compounds having pharmacological activity. Hence, our findings will demonstrate the influence of light stressor on growth and the phytochemical profile of the *D. forrestii.*

## 2. Results and Discussion

### 2.1. Light-Mediated Morphology and Growth

**Figure 1:**
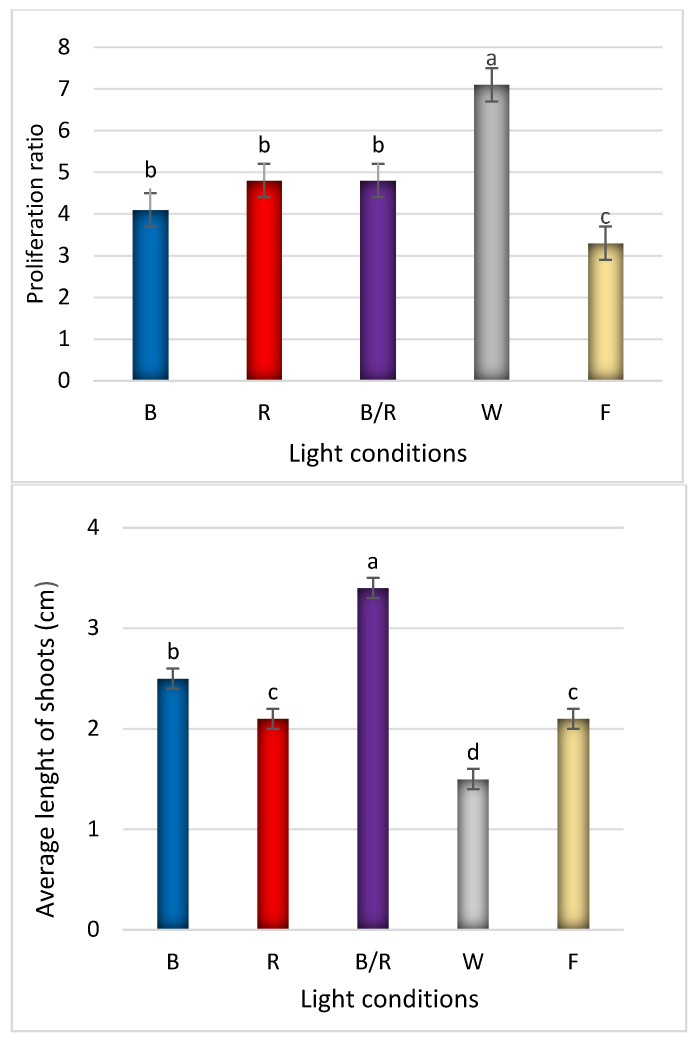
The effect of light conditions: (**B**) blue, (**R**) red, (**B/R**) blue/red, (**W**) white, (**F**) fluorescent on proliferation and length of *D. forrestii* shoots. The values represented means ± SE of three independent experimental replicates. The means marked with various letters for the same parameter were different at *p* < 0.05 according to one way ANOVA test followed by the post-hoc Tukey’s test.

**Figure 2:**
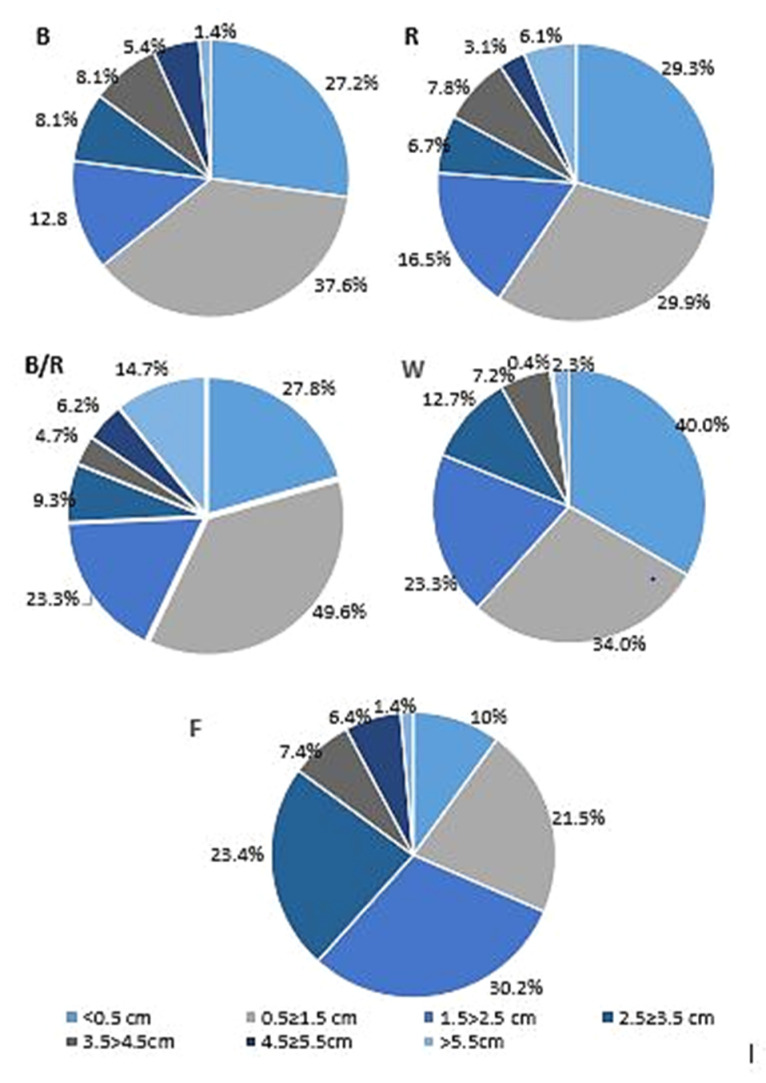
The effect of light conditions: (**B**) blue, (**R**) red, (**B/R**) blue/red, (**W**) white, (**F**) fluorescent on length shoots *D. forrestii* (based on the results from three independent experimental replicates).

**Figure 3:**
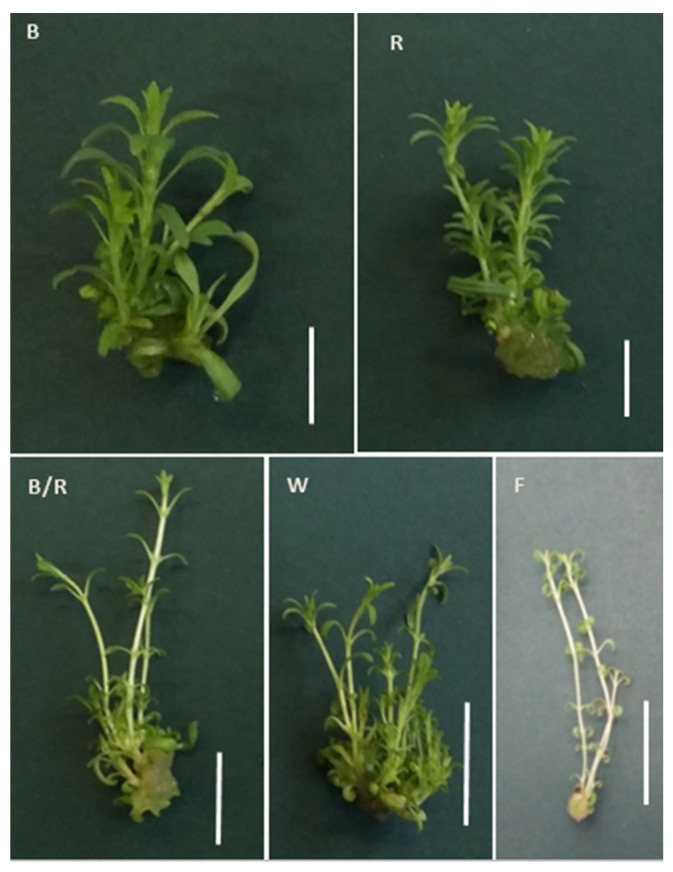
Morphology of *D. forrestii* shoots cultured under various light conditions: (**B**) blue, (**R**) red, (**B/R**) blue/red, (**W**) white, (**F**) fluorescent. Bar 1 cm.

**Figure 4:**
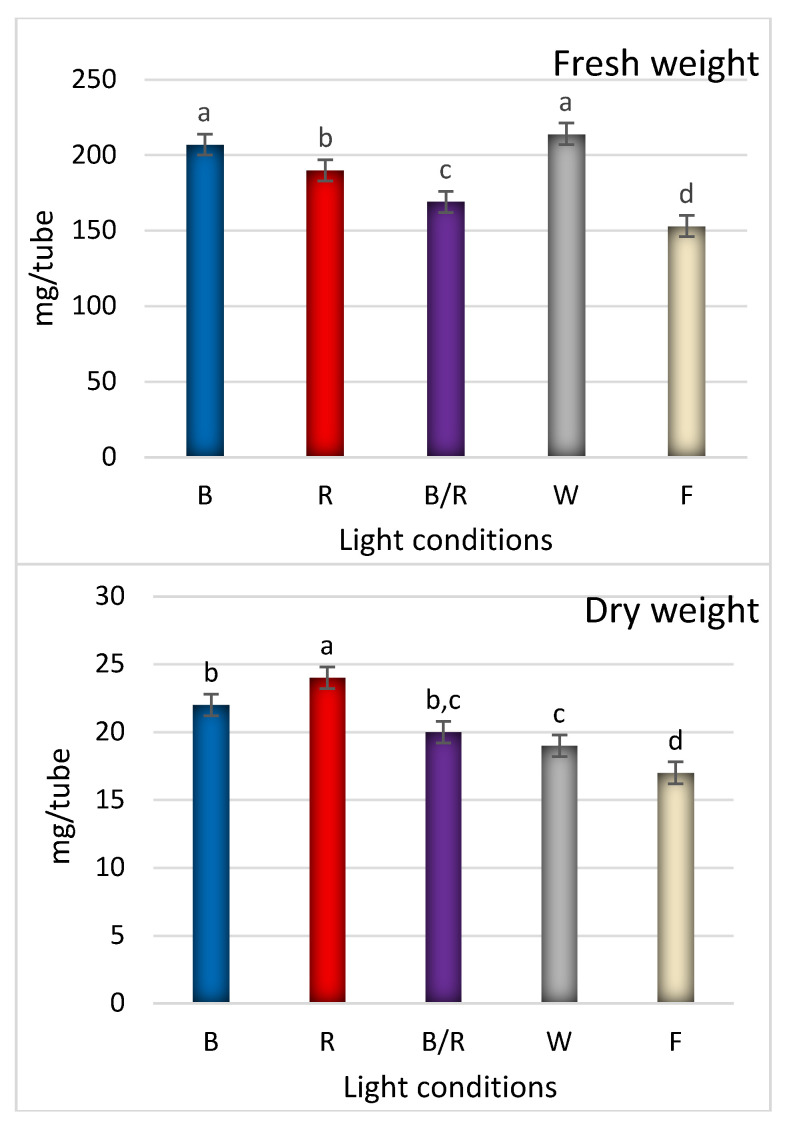
The effect of light condition: (**B**) blue, (**R**) red, (**B/R**) blue/red, (**W**) white, (**F**) fluorescent on biomass of shoots of *D. forrestii*. The values represented means ± SE of three independent experimental replicates. The means marked with various letters for the same parameter were different at *p* < 0.05 according to one-way ANOVA test followed by the post hoc Tukey’s test.

**Figure 5:**
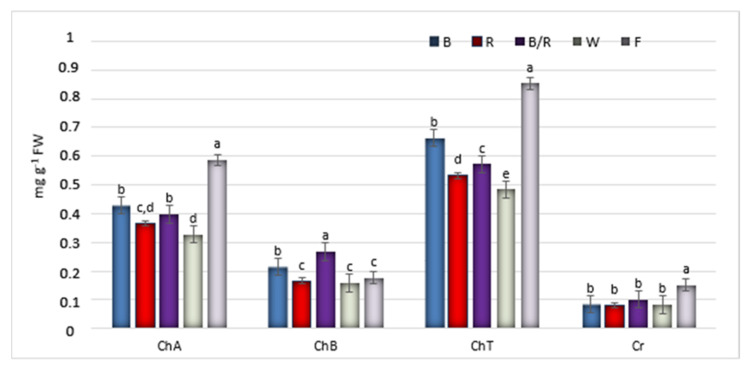
The effect of light conditions: (**B**) blue, (**R**) red, (**B/R**) blue/red, (**W**) white, (**F**) fluorescent on the photosynthetic pigments contents (mg g^−1^ FW) in shoots of *D. forrestii*. The values represented means ± SE of three independent experimental replicates. The means marked with various letters for the same parameter were different at *p* < 0.05 according to one way ANOVA test followed by the post-hoc Tukey’s test. (ChA) chlorophyll A, (ChB) chlorophyll B, (ChT) total chlorophyll, (Cr) carotenoids.

In the present study, fluorescent light, was chosen as a reference as it has played a dominant role as the supplemental lighting for greenhouse breeding for several decades. It encompasses a wider range of wavelengths, including both the visible and non-visible light spectrum (350–750 nm, although this range varies between reports) [12]. In our study, fluorescent lighting was found to result in the lowest rate of proliferation, with the shoots appearing fragile and demonstrating longer stems with long internodes and small, relatively short leaves (Figure 1, Figure 2 and Figure 3). Consequently, *D. forrestii* cultures also demonstrated the lowest biomass values (measured as fresh and dry weight) compared to the other LED sources (Figure 4). Surprisingly, the control demonstrated the highest photosynthetic pigment contents (ChA, ChT and Cr) (Figure 5); this may have been due to the fact that chlorophyll and carotenoid synthesis can be favoured by the wider and/or specific spectra provided by the fluorescent light with respect to the LED ones. In contrast to our present findings, illumination of *Saccharum officinarum* shoots with white LEDs resulted in greater pigment content than under other light treatments [13]; in our present study, LED white illumination resulted in the lowest levels of chloroplastid pigment production (Figure 5), highlighting a species-specific response to light stress.

All investigated growth parameters changed when LEDs were used to stimulate plant development. The most beneficial proliferation effect was observed in explants stimulated by white light (Figure 1); this may have been due to its spectral profile (Figure S1). A slightly lesser positive effect was demonstrated by the other LED variants, but no statistically significant differences were found between them. Finally, fluorescent illumination yielded the least response, giving the lowest shoot number. Interestingly, white light triggered the opposite reaction to fluorescent light with regard to proliferation: mean 7.1 ± 2.1 under white LED to 3.3 ± 1.2 shoots per explant by fluorescent-type lighting (Figure 1). Since the visible spectra of white LED and fluorescent light demonstrate quite significant overlapping, a response of *D. forrestii* may be due to species specific sensitivity to even slight spectra differences that results in significant change in the efficiency of bud induction. Hence, any generalisation to other species should be made with caution, as e.g., a clear increase in the number of shoots per explant was reported in *Vanilla planifolia* under both white LED and fluorescent light, as well as under blue/red LED lighting [14].

Further development of shoots, expressed as mean length, was most stimulated by combined blue/red light (3.4 ± 1.5 cm) compared to 2.1 ±1.1 cm under fluorescent light, followed by blue or red light alone (Figure 1 and Figure 2). The results are partially consistent with those of Silva et al. [13], indicating that this combination has a significant influence on shoot lengthening in sugarcane, in comparison to white LED; however, it was also found that the highest fresh weight and shoot multiplication ratio was stimulated by the blue/red light blend ratio [13]. Combined blue/red light was also found to have a positive influence on the shoot height in strawberry plants [15].

The white and blue LED regimes yielded an increase in fresh weight production; however, the highest dry biomass values were obtained under separate red and blue light conditions (Figure 4). Among all LED treatments, the total biomass ranged from 0.153 ± 0.020 g to 0.214 ± 0.030 g (FW) and from 0.017 ± 0.001 g to 0.024 ± 0.002 g (DW). It was found that the highest gain for FW was achieved by white and blue light equally, followed by red > blue/red > fluorescent light, while red light yielded the highest DW, followed by blue ≥ blue/red ≥ white > fluorescent light (Figure 4). It has been reported that blue and red wavelengths have a particularly strong effect on the opening and closure of stomata which control leaf gas exchange including both CO_2_ and the transpired water [16]. This transpiration changes may alter the water content in tissues, which can influence plant biomass, and the size and height of shoots [17]. The most spectacular change concerned the white light regime. While it results in the highest accumulation of fresh biomass, it does not effectively promote dry weight acquisition (Figure 4). Hence, it appears that white LED may be a better stimulator of tissue hydration and/or callus generation. Red and blue illumination turned out to display the most beneficial effect in dry biomass production; interestingly, they also induced internode shortening and leaf lengthening and widening (Figure 3 and Figure 4). For some species light-mediated hormonal regulation of plant growth was presented. The analysis of phytohormone transduction pathways revealed that some of the transcription factors were common for light and hormone signalling, hence light-mediated changes in the synthesis or activity of transcription regulators resulted in changes in phytohormone balance simultaneously. Namely, shoot elongation and root formation were promoted by auxin activity enhanced by blue light. The blue light caused an increase in the activity of indole-3-acetic acid oxidase, the enzyme that stimulates the biosynthesis of auxin [18]. However, the range and direction of response may vary depending on species and light spectrum. For example, Petunia displayed higher biosynthesis of gibberellins (and subsequent shoot elongation) under blue light conditions than when red light was used [19]. However, again this is not a general rule in LED-mediated plant breeding; for example, studies on grapes indicate that red LEDs induced the highest shoots with longer internodes [20].

Among tested LED sources the chloroplastid pigments reached maximum values under blue and blue/red treatments (Figure 5). The red LED operated antagonistically during pigment synthesis. Our data confirm previous findings that a blue source of light is a significant factor for chlorophyll induction, while red decreases its level [8]. Blue illumination has also been reported to positively influence chlorophyll synthesis and chloroplast development in studies on *Chrysanthemum* and *Tripterospermum japonicum* [21,22].

### 2.2. Bioactive Compound Production

**Figure 6:**
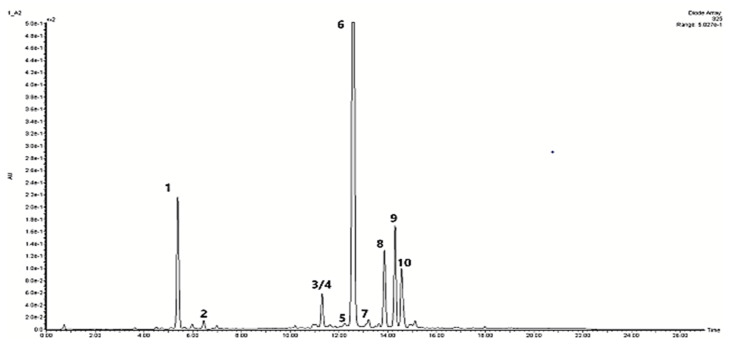
HPLC chromatogram of hydromethanolic extract from shoot culture of *D. forrestii* grown in blue light Peak numbers correspond to those in Table 1. The detection wavelength was set at 325 nm. (1) Chlorogenic acid, (2) Caffeic acid, (3/4) Salvianolic acid I/H and Salvianolic acid E, (**5**) Dicaffeoylquinic acid, (6) Rosmarinic acid, (7) Lithospermic acid, (8) Salvianolic acid B, (9) Apigenin *p*-coumarylrhamnoside, (10) Metyl rosmarinate.

The extracts from the cultivated shoots contained ten examined phenolic compounds, including nine phenolic acid derivatives and one flavonoid (Table 1, Figure 6). All these compounds were previously detected in the extract of *D. forrestii* shoots grown in vitro [5,7].

The total phenolic compound content ranged from 13.824 ± 1.181 to 20.018 ± 0.801 mg/g DW, depending on LED treatment (Table 1), which indicates that light is not only an important factor for photosynthesis, but for other physiological mechanism involved in the biosynthesis of SM [23]. HPLC analysis indicates that white LEDs were less favourable for the production of majority of phenolic acids than the other light conditions (Table 1). The quantitatively dominant metabolite was rosmarinic acid (RA); it was found to be present at the highest levels in the control shoots (11.461 ± 0.759), followed by those grown under red LEDs (10.896 ± 0.180). Although these two values were not significantly different, they were more than twice as high as those in the shoots grown under white LEDs (4.942 ± 0.314 mg/g d.w.) (Table 1). Other studies have also demonstrated a disadvantageous effect of white light on production of shikonin derivatives in *Lithospermum erythrorhizon* [24] or tropane alkaloids in *Hyoscyamus muticus* [25]. Elsewhere, control fluorescent lamp light was found to stimulate the greatest accumulation of gallic acid in in vitro cultures of *Myrtus communis* compared to all LED variants (red, blue, red/blue) [26].

However, all LED treatments were found to increase biosynthesis of polyphenolic acids with more complex molecular structure, such as salvianolic acids, in *D. forrestii* shoots (Table 1). Salvianolic acid B (SalB), a dimer of RA, was the second most quantitatively dominant compound in the studied extracts, with amounts ranging from 3.608 ± 0.144 mg/g d.w. in shoots under red LEDs to 2.884 ± 0.278 and 2.842 ± 0.251 mg/g d.w., respectively, under white and mixed red/blue LEDs; in contrast, it was present at only 0.289 ± 0.020 mg/g d.w. in the control shoots (Table 1). Previous studies have shown that SalB can inhibit platelet aggregation and adhesion, which has great significance for the prevention of cardiovascular diseases [27]. SalB has been found to reduce oxidative stress by protecting animal cells from peroxidation and free radical damage [28]. Our results indicate that SalB content was always higher under LED treatments, however LED did not stimulate the increase in RA content. Conversely, it has previously been reported that LED treatment stimulated RA synthesis but did not catalyse its conversion to SalB in *S. miltiorrhiza* hairy roots; the authors hypothesized that SalB is not obtained by conversion from RA, but from danshensu via the tyrosine-derived pathway [29]. This would explain why changes in SalB production were not related to changes in RA production.

Also, significantly higher combined salvianolic acid H/I and E content was observed during the growth under LEDs than under fluorescent lamps (Table 1). The compound contents peaked in shoots under mixed red/blue treatment (1.921 ± 0.093 mg/g d.w.), this being 2.5-fold greater than in control conditions.

Combined red/blue and blue light alone yielded the greatest production of chlorogenic acid (Table 1), while red light significantly reduced it. In a previous study of *Peucedanum japonicum* callus culture [30], optimal chlorogenic acid production (3.4 mg/g d.w.) was obtained for mixed light, albeit in another combination (3Red3Blue3Infra-red); in contrast, chlorogenic acid production fell under blue or red light alone, or a mixture of red and blue, with a predominance of red light, (0–0.09 mg/g d.w.) compared to shoots grown under white light (0.44 mg/g d.w.). Similar results were reported for *Ruta graveolens*: red light inhibited chlorogenic acid biosynthesis compared to other LED treatments [31]. However, red light induced maximum production of caffeic acid in *Ocimum bassilicum* [32] and cinnamic acid in *Ruta graveolens* [31].

Blue LEDs were found to be extremely beneficial for the production of the flavonoid, apigenin *p*-coumarylrhamnoside in shoot culture of *D. forrestii* (Table 1). A high level has been reported in *Agrobacterium rhizogenes* transformed shoots, but only traces were found in the young wild type shoot culture [5,7,33]. Apigenin *p*-coumaryl rhamnoside biosynthesis appears to be particularly sensitive to lighting conditions: blue LED treatment yielded twice as much as other light conditions. Blue light was also most beneficial for production of the flavonoid glycosides cynaroside, quercitrin and rutoside in shoot cultures of three *Aronia species* [34].

In summary, our findings confirm that blue and red light are needed for optimal total phenol production (20.018 ± 0.801 and 19.161 ± 1.36 mg/g d.w., respectively) in the shoots of *D. forrestii*; however, the profile of the individual compounds differed between these two treatments (Table 1). This clearly proves that different light conditions stimulate the individual polyphenolic compound biosynthesis pathway influencing only its selected nodal points (the nodal points of the polyphenol metabolism pathway are catalysed by one specific enzyme, the production of which is associated with changes in the expression of a single gene that could be influenced by different light spectra; it is likely that only selected nodal points are activated/inhibited under a given light regime changing the synthesis of a specific compound without significantly influencing production of the others). This is likely connected with the influence of light on the expression of biosynthetic genes via photoreceptors [35]. Blue light often promotes the accumulation of total phenolic compounds in in vitro cultures. Such an effect has been described for the biosynthesis of verbascoside in *Verbena officinalis* [36] or *Scutellaria lateriflora* [37] or total phenols in the leaf extracts of *Rehmannia glutinosa* [36]. On the other hand, the total flavonoid content in *R. glutinosa* and in *Ocimum bassilicum* culture increased most intensely under red LEDs [32,38]. Similarly, myricetin was clearly promoted by red light in in vitro cultures of the *Myrtus communis* [21].

### 2.3. Antioxidant Response

**Figure 7:**
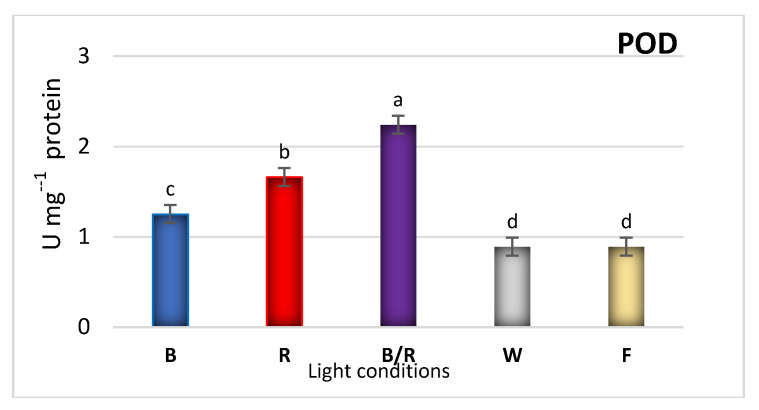

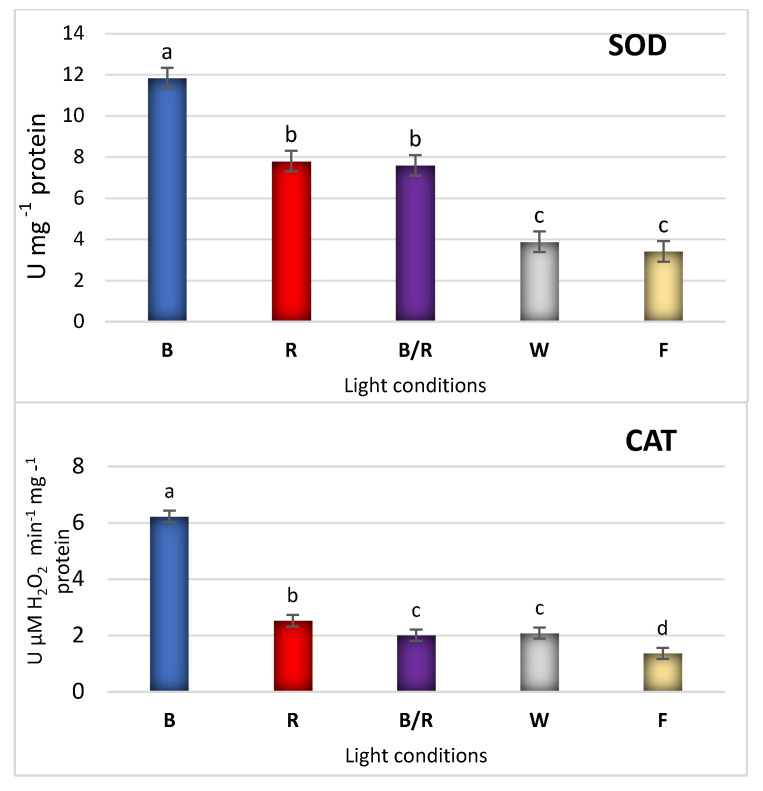
The effect of light conditions: (**B**) blue, (**R**) red, (**B/R**) blue/red, (**W**) white, (**F**) fluorescent on the antioxidant enzyme (POD—peroxidase, SOD—superoxide dismutase, CAT—catalase) activities in shoots of *D. forrestii*. The values represented means ± SE of three independent experimental replicates. The means marked with various letters for the same parameter were different at *p* < 0.05 according to one way ANOVA test followed by the post-hoc Tukey’s test.

The fast responses of antioxidant enzyme activities such as POD, CAT and SOD, and SM production are the important strategies for reducing ROS formed during stress reactions [39]. High phenolic content enhances the antioxidant property of the plant, because phenols are capable of scavenging free radicals and preventing oxidative damage. As polyphenol production has been found to change when plants were exposed to light, changes in the antioxidant potential of a culture can also be expected. Furthermore, in *D. forrestii* shoots, the LED wavelengths were found to not only enhance the polyphenol content, but also the activities of antioxidant enzymes (Figure 7).

The blue LED treatment dramatically increased CAT (4.5-fold) and SOD activities (3.5-fold) compared to shoots under fluorescent lamps (Figure 7). The results were similar to these obtained for leaves of *Rehmannia glutinosa*, in which blue LED treatment demonstrated the greatest influence on enzymatic antioxidant mechanism [33]. Blue light treatment also increased POD activity in shoots of *D. forrestii*, but the highest enzyme activity level was found under mixed light (Figure 7).

**Figure 8:**
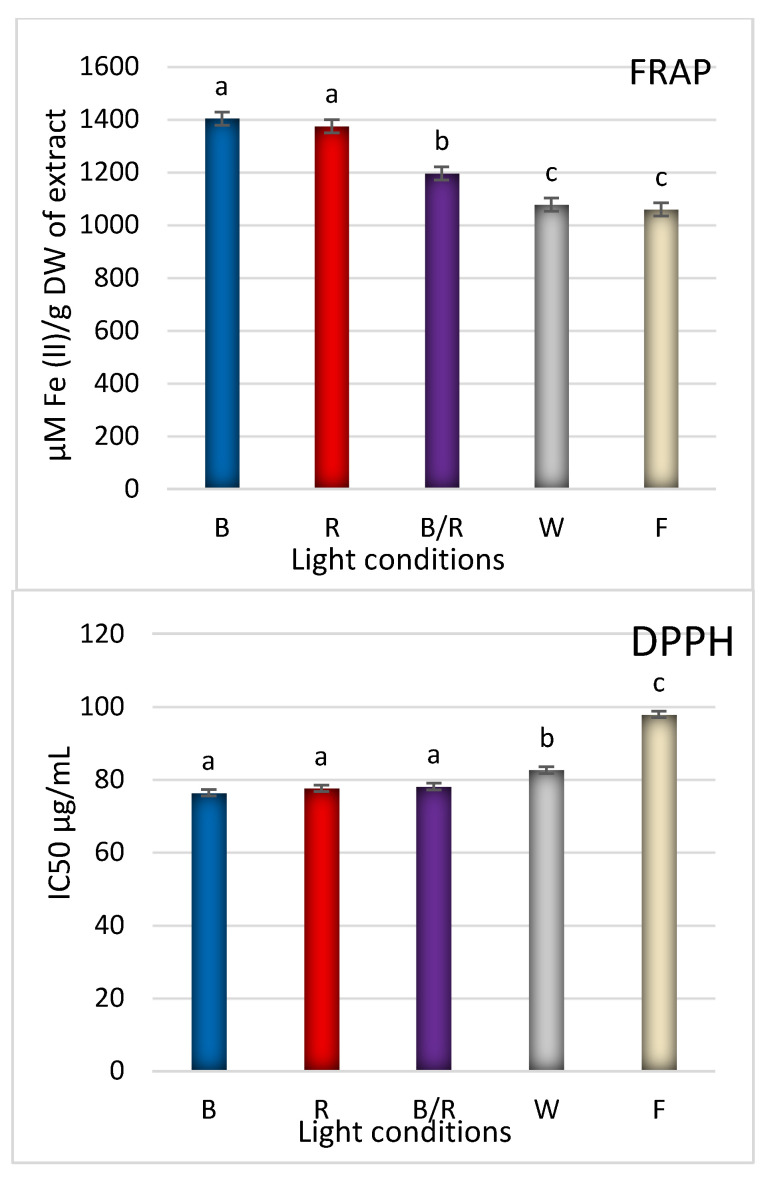
The effect of light conditions: (**B**) blue, (**R**) red, (**B/R**) blue/red, (**W**) white, (**F**) fluorescent on the antioxidant capacity (FRAP---ferric reducing antioxidant power, DPPH---1,1-diphenyl-2-picrylhydrazyl) of hydromethanolic extract from shoots of *D. forrestii*. The values represented means ± SE of three independent experimental replicates. The means marked with various letters for the same parameter were different at *p* < 0.05 according to one way ANOVA test followed by the post-hoc Tukey’s test.

The highest iron reduction capacity was observed for *D. forrestii* shoots grown under red light, followed by blue (Figure 8) (no statistical differences); this might be due to fact that these treatments are eliciting the highest total phenol content, especially phenolic acids (including SalB and RA). Shoots treated with white LEDs or F light show a significantly weaker ability to reduce iron (Figure 8); the two treatments demonstrated comparable iron reduction potential, although the white LED treatment yielded a three-fold lower content of RA (Table 1). This reflects the strong antioxidant activity of SalB, the content of which was significantly higher in the shoots under red and blue light—the conditions that yielded the highest level of iron reduction capacity. Hence, extrapolating this result, if in the case of white LED treatment, the RA content was low and SalB content was high, there is likely that SalB is responsible for significant antioxidant potential. SalB is known as natural antioxidant obtained from *S. miltiorrhiza* and demonstrated strong potential in scavenging O_2_^-^ and OH^-^ and inhibiting lipid peroxidation of microsomes [40]. Chen et al. [41] showed that salvianolic acid B has a higher radical scavenging capacity (IC_50_ value = 8.8 μM than other phenolic acids e.g., chlorogenic acid (IC_50_ value = 27.5 μM).

Similarly, among all light treatments, the shoots cultivated under red light demonstrated the highest DPPH radical scavenging potential, followed by the blue treatment (no statistical differences) (Figure 8).

The results of our studies: the increased enzyme activities and antioxidant assay findings are consistent with the fact that the cultures grown under red and blue light produced the highest amounts of antioxidant compounds. This could indicate that the plant tried to adapt to these specific spectral ranges. A particularly strong direct relationship was found between polyphenol content in *D. forrestii* culture and the results of the iron reduction test (*r* = 0.91) and superoxide dismutase activity (r = 0.88) (Table 2). A positive correlation between antioxidant potential and phenolic content has been also reported for callus culture of *Lepidium sativum* [42], *Basella rubra* [43] or *Fagonia indica* [44].

## 3. Conclusions

Plant secondary metabolites are usually studied in the context of their role in plant defence against abiotic and biotic stresses. Little data is available regarding the correlation between the culture light conditions and the mechanisms underlying the physiological secondary metabolism in *D. forrestii*. Our findings indicate that under controlled in vitro conditions, manipulation of light quality could induce significant changes to physiological and biochemical responses of *D. forrestii* shoot culture. Optimum biomass accumulation and shoot micropropagation ratio were recorded under blue light, as was SM accumulation. Moreover, it has been observed that light-stimulated *D. forrestii* shoots display specific stress-derived phytochemical profiles leading to more effective synthesis of pharmacologically-active compounds. Maximum total polyphenolic acid levels were achieved by exposure to blue light treatment followed by red LEDs, and this was strongly correlated with an enhancement of antioxidant capacity. Hence, light stress may turn out to be a simpler alternative to metabolic engineering offering new perspectives for improving the production of such valuable compounds.

## 4. Materials and Methods

### 4.1. Plant Material

The in vitro culture shoots of *D. forrestii*, were initiated from seeds obtained from Parco Nazionale Gran Paradiso, Valnontey (Italy). The optimization of shoot growth conditions and bioactive compound production are described by Weremczuk-Jeżyna et al. [5]. In the present experiment, nodal segments 1 cm in length were used as explants. The explants were placed on MS (Murashige and Skoog) [45] agar (0.7%) medium containing 0.5 mg/L BAP and 0.2 mg/L IAA. Shoot cultures were kept at 26 ± 2 °C under a 16 h photoperiod provided by different light conditions. After five weeks of culture, the number of shoots or buds per explant was recorded, as was their length, fresh (FW) and dry weight (mg/tube) (DW). Each experiment including at least 20 explants was repeated three times.

### 4.2. Light Conditions

In this study, four LEDs were used: the lamps emitted blue light (430 nm), red light (670 nm), a combination of red and blue lights (70%:30%) and white light (430–670 nm) (PXM Sp. (Niepołomice, Poland). Spectral characterization of the tested LED lamps was made using a BTS256-LED Tester (Gigahertz-Optik, Germany). Fluorescent light emitted by cool white fluorescent lamps was used as a control. The light intensity was the same for all light sources (40 μM m^−2^ s^−1^). The spectral characterisation of LEDs showed Figure S1.

### 4.3. Analysis of Photosynthetic Pigments

The samples were prepared with 80% acetone by macerating 0.2 g of FW of the shoots according to method described by Oren et al. [46]. The photosynthetic pigments content was determined spectrophotometrically (UV-1800 UV/VIS Spectrophotometer (Beijing, China). The absorbance of samples was indicated at wavelengths: chlorophyll a (664 nm), chlorophyll b (647 nm) and carotenoids (470 nm) [47]. The level of pigments were expressed as mg g^−1^ FW.

### 4.4. Extraction of Phenolic Acids

Lyophilized and powdered shoots (100 mg) were first sonicated with 15 mL chloroform using a UD-20 ultrasonic disintegrator (15 mL). After filtration, the defatted samples were sonicated for 15 min with 80% (*v*/*v*) aqueous methanol (25 mL) at 40 °C in an ultrasonic bath, and then twice in 10 mL of the same solvent for 15 min. The extracts were filtered and evaporated to dryness under reduced pressure. The residue was dissolved in methanol (2 mL) and centrifuged at 18,000 rpm for three minutes, and the supernatant was analyzed by HPLC.

### 4.5. Determination of Phenolic Acids Content

A quantitative analysis was performed using a Waters HPLC system consisting of a binary HPLC pump (Waters 2545), a diode array detector (Waters 2998) and an auto sampler (Waters 2767). MassLynx software (version 4.1) was used for instrument control and data acquisition. The analysis was performed on a XBridge C18 OBD column (4.6 mm × 100 mm) with a particle size of 5μm.The mobile phase (A) was 0.1% trifluoroacetic acid in water and the mobile phase B was 0.1% trifluoroacetic acid in acetonitrile. The following gradient system was used for the analysis: 0–20 min 5–50 solvent B, 20–21 min 50% solvent B, 21–22 min 50−5% solvent B, 22–27 min 5% solvent B. The flow rate was 1.6 mL/min. UV spectra were recorded over range of 190–700 nm, chromatograms were acquired at 325 nm. The injection volume was arranged as 4 μL. The compounds were identified by comparison of their retention times and UV spectra with those of the standard compounds and/or literature data [5,7]. For quantitative analysis, individual standard calibration curves were constructed based on the area peaks. Standards: chlorogenic acid, caffeic acid, RA were obtained from Sigma-Aldrich, Germany and apigenin-7-*O*-glucoside and SalB from Extrasynthese, France. Compounds, for which pure standards were not available, were quantified according to the calibration curve of similar standards.

### 4.6. Determination of Activities of Antioxidant Enzymes

The extracts needed to analyze peroxidase (POD), catalase (CAT) and superoxide dismutase (SOD) activity were obtained by grinding (in 4 °C) fresh biomass of shoots (0.5 g) with 4 mL phosphate buffer (pH = 7.5) with the addition of 0.5 mM EDTA. Next, the mixture was centrifuged (12,000 rpm for 10 min) and supernatant was taken for antioxidant enzyme activity assay by spectrophotometry (UV-1800, UV/VIS Spectrophotometer). The CAT activity was determined at 240 nm and results expressed as units mg^−1^ of protein (U = 1 µM of H_2_O_2_ reduction min^−1^ mg^−1^ protein) [48]. The SOD activity was evaluated according to Giannopolitis and Reis [49]: the ability to inhibit reduction of nitro blue tetrazolinum (NBT) (Sigma-Aldrich, Darmstadt, Germany) and the absorbance of the samples was determined at 560 nm [50]. SOD activity was expressed as enzyme units per mg of protein (U mg^−1^ protein). The POD activity was determined by the increase in absorbance at 470 nm due to guaiacol (Sigma-Aldrich, Germany) oxidation [51]. The activity was reported as U mg^−1^ protein. Bovine serum albumin (Sigma-Aldrich, Germany) was used as standard protein. Protein concentration was determined according to Bradford [52].

### 4.7. Antioxidant Assays

Antioxidant analysis was performed using hydromethanolic extracts obtained from 1 g dried, lyophilized and powdered plant material. The same extraction procedure was used as for the phytochemical analysis. The antioxidant potential of the extracts from transformed *D. forrestii* shoots grown under different light conditions was determined using two antioxidant tests: ferric reducing antioxidant power (FRAP) and 1,1-diphenyl-2-picrylhydrazyl (DPPH) radical assays.

For the FRAP assay, antioxidant activity was determined spectrophotometrically against a calibration curve of ferrous sulfate; absorbance was measured at 595 nm and expressed as μM Fe(II) g^−1^ DW of extract [53]. The radical scavenging activity (DPPH test), was determined according to [54]; absorbance was measured after 30 min at 517 nm, and antiradical activity was expressed as IC_50_ value (μg mL^−1^). IC_50_ value is the concentration of the sample demonstrating 50% of maximum absorption.

### 4.8. Statistical Analysis

The data were presented as means ± SE (standard error). All the aforementioned tests were repeated three times. All estimated values including values of means, standard deviations, standards errors, EC_50_ values, correlation coefficients between polyphenolic compound content and antioxidant activity as well as enzyme antioxidant activity were calculated using MS-Excel (Microsoft Sp. Z o. o., Warsaw, Poland). The results were analyzed using the one-way ANOVA test and multivariate analysis of variance, followed by the Tukey’s *post-hoc* test. The significance level for all calculations was set at *p* < 0.05. All tests were performed using STATISTICA 10.0 software (STATSoft, Krakow, Poland).

## Figures and Tables

**Table 1:** The effect of light conditions: blue, red, blue/red, white, fluorescent on accumulation of phenolic compounds in *D. forrestii* shoot culture. The values represented means ± SE of three independent experimental replicates.

Peak No.	Compound	Compound Content [mg/g DW] under Different Light Treatments				
		Blue	Red	Red/Blue	White	Control
1	Chlorogenic acid	2.001 ± 0.065 ^a^	1.021 ± 0.125 ^c^	2.043 ± 0.045 ^a^	1.839 ± 0.143 ^b^	1.086 ± 0.101 ^c^
2	Caffeic acid	0.096 ± 0.002 ^b^	0.040 ± 0.003 ^d^	0.049 ± 0.005 ^cd^	0.054 ± 0.025 ^c^	0.133 ± 0.011 ^a^
¾	Salvianolic acid I/H Salvianolic acid E	1.364 ± 0.050 ^c^	1.196 ± 0.135 ^c^	1.921 ± 0.093 ^a^	1.513 ± 0.151 ^b^	0.777 ± 0.066 ^d^
5	Dicaffeoylquinic acid	0.049 ± 0.003 ^b^	0.043 ± 0.004 ^b^	0.043 ± 0.003 ^b^	0.06 ± 0.006 ^a^	0.053 ± 0.007 ^a,b^
6	Rosmarinic acid	9.187 ± 0.320 ^b^	10.896 ± 0.810 ^a^	6.833 ± 0.591 ^c^	4.942 ± 0.314 ^d^	11.461 ± 0.759 ^a^
7	Lithospermic acid	0.492 ± 0.016 ^b^	0.151 ± 0.004 ^d^	0.552 ± 0.016 ^a^	0.546 ± 0.045 ^a,b^	0.414 ± 0.028 ^c^
8	Salvianolic acid B	3.285 ± 0.253 ^a,b^	3.608 ± 0.144 ^a^	2.842 ± 0.251 ^b^	2.884 ± 0.278 ^b^	0.289 ± 0.020 ^c^
9	Apigenin *p*-coumarylrhamnoside	2.448 ± 0.053 ^a^	1.171 ± 0.084 ^c^	1.632 ± 0.110 ^b^	1.180 ± 0.140 ^c^	1.305 ± 0.061 ^c^
10	Metyl rosmarinate	1.096 ± 0.028 ^a^	1.035 ± 0.013 ^b^	1.137 ± 0.045 ^a^	0.806 ± 0.063 ^c^	0.551 ± 0.028 ^d^
Total phenol content		20.018 ± 0.801 ^a^	19.161 ± 1.360 ^a,b^	17.052 ± 1.170 ^b,c^	13.824 ± 1.181 ^d^	16.069 ± 1.090 ^c,d^

The means marked with various letters for the same parameter were different at *p* < 0.05 according to one way ANOVA test followed by the post-hoc Tukey’s test.

**Table 2:** Correlation coefficient between antioxidant activity and phenolic content in *D. forrestii* shoot culture under various light conditions.

*r*	FRAP	DPPH	POD	SOD	CAT
Total phenol content	0.91	−0.67	0.40	0.88	0.70

## Data Availability

The data presented in this study are available from the corresponding author on request.

## Supplementary Materials

The following are available online at https://www.mdpi.com/article/10.3390/ijms22157965/s1. Figure S1. The spectral characterisation of used LEDs: (A) blue LED (430 nm), (B) red LED (670 nm), (C) white LED (390–760 nm). Red/blue LED was mixed red (70%) and blue (30%) LED.
