# Supplementary material for: The Protective Function and Modification of Secondary Metabolite Accumulation in Response to Light Stress in Dracocephalum forrestii Shoots
Source: Int J Mol Sci. 2021 Jul 26;22(15):7965. doi: 10.3390/ijms22157965 (PMC8347274; doi:10.3390/ijms22157965)
Supplement: Supplementary file 1 [file ijms-22-07965-s001.zip › ijms-1233650-supplementary.pdf]

Supplementary Material

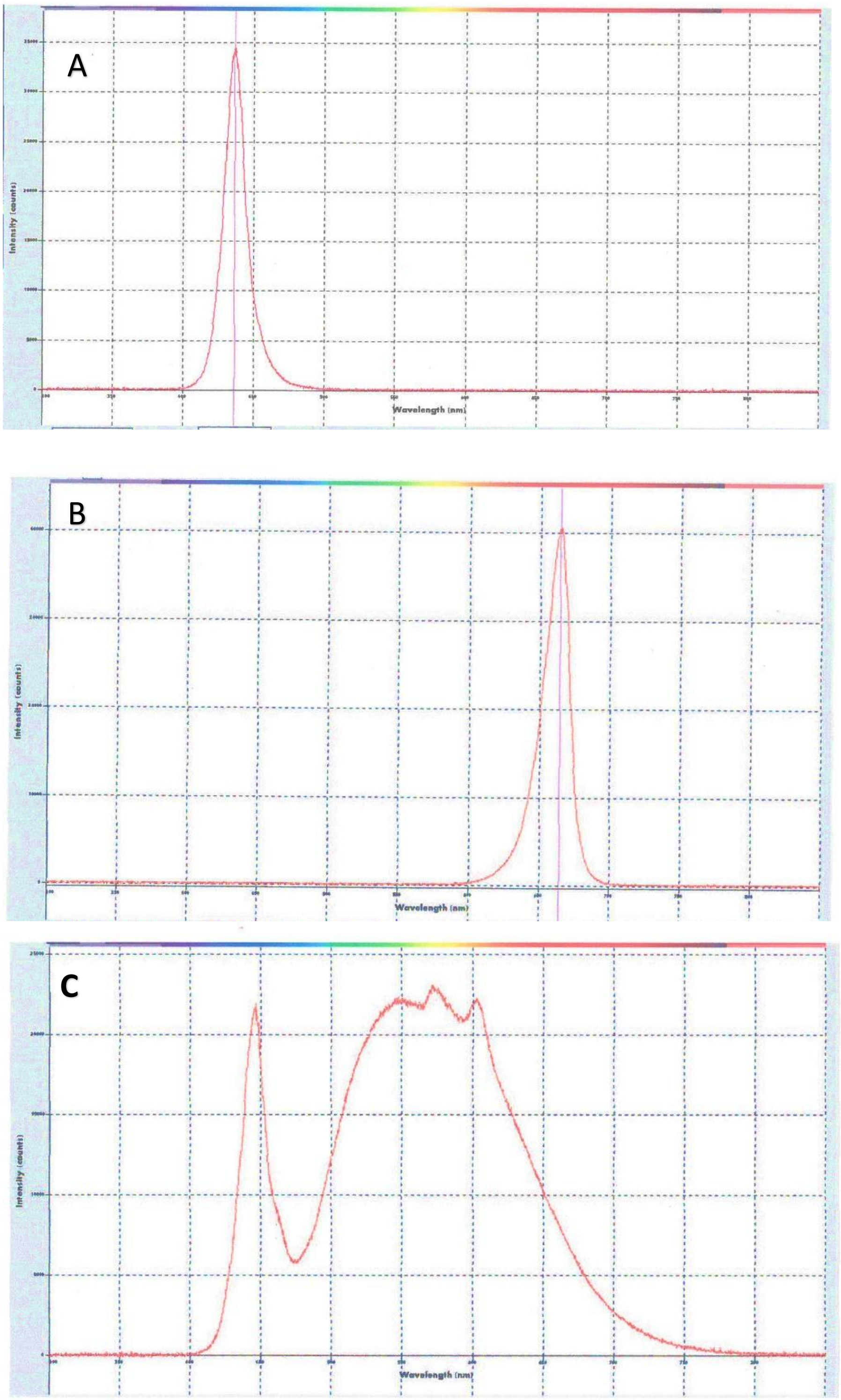

Figure S1. The spectral characterisation of used LEDs: A) blue LED (430 nm), B) red LED (670 nm), C) white LED (390–760 nm). Red/blue LED was mixed red (70%) and blue (30%) LED.
